# New Species-Specific Real-Time PCR Assays for *Colletotrichum* Species Causing Bitter Rot of Apple

**DOI:** 10.3390/microorganisms12050878

**Published:** 2024-04-27

**Authors:** Diana J. McHenry, Srđan G. Aćimović

**Affiliations:** Plant Pathology Laboratory, Alson H. Smith Jr. Agricultural Research and Extension Center, School of Plant and Environmental Sciences, Virginia Polytechnic Institute and State University, Winchester, VA 22602, USA

**Keywords:** *Colletotrichum*, apple bitter rot, real-time PCR, pathogen diagnostics, rapid detection

## Abstract

Bitter rot of apple is an economically important worldwide disease caused by different *Colletotrichum* species, depending on many factors such as climate, geography, other hosts, and crop management practices. Culture, morphology, and single-locus sequencing-based methods for identifying the *Colletotrichum* species are severely limited in effectiveness, while the multilocus sequence typing methods available for delineating species are costly, time-intensive, and require high expertise. We developed species-specific hydrolysis probe real-time PCR assays for the following nine *Colletotrichum* species causing bitter rot in the Mid-Atlantic U.S.A.: *C. fructicola*, *C. chrysophilum*, *C. noveboracense*, *C. gloeosporioides* s.s., *C. henanense*, *C. siamense* and *C. theobromicola* from the *C. gloeosporioides* species complex, and *C. fioriniae* and *C. nymphaeae* from the *C. acutatum* species complex. After searching 14 gene regions, we designed primers and probes in 5 of them for the nine target species. Four primer–probe set pairs were able to be duplexed. Sensitivity tests showed as little as 0.5 pg DNA were detectable. These real-time PCR assays will provide rapid and reliable identification of these key *Colletotrichum* species and will be critically important for studies aiming to elucidate their biology, epidemiology, and management on apples as the number one produced and consumed tree fruit in the U.S.A.

## 1. Introduction

Apple bitter rot is a severe disease leading to direct fruit losses ranging from 2 to 100% [1,2,3,4,5]. The economic impacts of bitter rot in the U.S.A. are estimated to be between $300 and $400 million annually. Wet and warm weather conditions favor bitter rot infections during the late spring and summer. Typical brown circular and flat to sunken lesions on apple fruit can occur both in the orchard and postharvest in storage [6,7,8].

This complex disease is caused by multiple fungal species in the genus *Colletotrichum*. There are three species complexes within *Colletotrichum* with pathogens infecting apple and pear fruits as follows: (1) acutatum species complex (CASC), (2) gloeosporioides species complex (CGSC), and (3) boninense species complex [9,10,11]. Over the last 8 years, efforts in the Mid-Atlantic U.S.A. have led to identifying the following nine species as causal agents of apple bitter rot: *C. chrysophilum*, *C. fructicola*, *C. noveboracense*, *C. siamense*, *C. theobromicola*, *C. henanense* and *C. gloeosporioides* sensu stricto (s.s.) from CGSC, and *C. fioriniae* and *C. nymphaeae* from CASC. *C. chrysophilum* is also the primary cause of the leaf form of this disease on apples called Glomerella leaf spot which, in Southeastern U.S.A. and several South American countries, can rapidly defoliate apple trees [12,13,14,15]. In grapes, often grown close to apples, *Colletotrichum* causes ripe rot disease. Up to 20 *Colletotrichum* species worldwide have been reported to be infecting grape berries, causing losses [16,17].

The *Colletotrichum* genus, encompassing over 200 known species, presents a challenge in taxonomy due to its high genetic variability. Initial attempts at classification relied on morphological characteristics, but issues arose from the lack of standardized culturing and ambiguous traits that were insufficient for quick differentiation. Various approaches, such as secondary metabolite profiling, pathogenicity testing, cross-mating, physiological studies, carbon source utilization, and molecular phylogeny, were employed to characterize the *Colletotrichum* species. However, a singular conserved DNA barcode proved elusive, with markers like GAPDH, ACT, CHS, HIS3, and TUB2 initially considered [18]. Subsequent studies revealed the limitations of a single DNA barcode marker for all *Colletotrichum* spp., prompting a multilocus approach. Vieira et al. [19] reported that a concatenated phylogeny with additional intergenic markers like APN2/MAT-IGS, GAP2-IGS, and APN2 differentiated the *C. gloeosporioides* complex, while HIS3, GAPDH, and TUB2 distinguished the *C. acutatum* species complex. This and other approaches uncovered novel species on apples like *C. noveboracense* [10] and *C. orientalis* [20] and identified a previously described species on bananas, *C. chrysophilum* [21], causing bitter rot on apples [10,22]. Ongoing efforts utilize whole genome sequencing and various descriptive genomics facets to refine the *Colletotrichum* spp. taxonomy [23]. Nevertheless, all these differentiation efforts require high expertise and are an obstacle for rapid and cheap pathogen identification for the facilitation of species-specific field or storage sample investigations and treatment, particularly for apple diseases caused by the *Colletotrichum* species.

Accurate and rapid identification of the *Colletotrichum* species causing apple bitter rot is vital for *Malus* resistance breeding [23,24]. It is also essential for the development of effective control strategies while minimizing risks for single-site fungicides resistance in these pathogens [5,11,25,26]. Furthermore, fast detection of the *Colletotrichum* spp. in early, untypical spots on flowers and leaves or rot symptoms on apple fruit would lead to more timely decisions in implementing effective management options. Finally, in North Carolina, Villani et al. [27] found that symptoms of apple bitter rot, predominantly caused by the species in the CGSC, are indistinguishable from rots caused by other fungal pathogens, e.g., *Botryosphaeria obtusa*, B. dothidea, *Botrytis cinerea,* and others. Furthermore, late fruit infections by *Colletotrichum*, just before apple harvest, lead to indistinguishable rot symptoms from the ones caused by other postharvest pathogens, expressing when fruit are prepared for or placed in cold storages. This necessitates rapid diagnostic assays to identify the *Colletotrichum* species as the primary cause of rot and distinguish it from other less invasive rots.

Molecular detection assays have been developed for many *Colletotrichum* species using various genes [28,29,30,31,32,33,34,35,36,37,38,39]. In several cases, only a few non-target *Colletotrichum* species were used in the specificity testing of the assay; often, only species found on the same host plant in the same geographical region were included [29,37,39]. This is a straight-forward, appropriate strategy for those small host–pathogen-geography systems, but it can lead to non-specific amplification or false positives when the assay is used outside that system. A PCR primer set could be species-specific for species A when tested among only species A, B, and C; the same primer set may also unintentionally amplify species Y and Z. In addition, given the uncertainty around past species delineation within the genus, and how closely related many *Colletotrichum* species are, it is important to include as many related species and as many isolates within each species as is feasible when testing new molecular detection assays.

In recent studies of the Mid-Atlantic *Colletotrichum* on apples, the most common species were *C. fioriniae* and *C. chrysophilum* [10,11,22]. Other species occurring on apple were *C. fructicola*, *C. noveboracense*, *C. siamense*, *C. henanense*, *C. nymphaeae*, *C. gloeosporioides* s.s., and *C. theobromicola* [10,11,22]. In the northern Mid-Atlantic, *C. fioriniae* and *C. chrysophilum* were most common, while *C. fructicola* was dominant in the south.

The aim of this study was to develop species-specific hydrolysis probe real-time PCRs for molecular detection and identification of the following nine causal agents of bitter rot on apple in the Mid-Atlantic U.S.A.: *C. chrysophilum*, *C. fioriniae*, *C. fructicola*, *C. gloeosporioides* s.s., *C. henanense*, *C. noveboracense*, *C. nymphaeae*, *C. siamense*, and *C. theobromicola*.

## 2. Materials and Methods

### 2.1. Fungal Isolates, Strains, Culture Media, and DNA Extraction

A total of 88 *Colletotrichum* isolates in 16 species, plus 10 other fungal species, apples, and grapes were used in this study (Table 1). Isolates were grown on PDA at 25 °C for DNA extraction, which was performed on mycelia with a DNeasy Plant Mini Kit (QIAGEN, Germantown, MD, USA). DNA quality was determined via gel electrophoresis. Isolates were previously identified to the species level [3,10,22,23,40,41,42,43,44,45,46,47,48,49].

### 2.2. Primer and Probe Design, Specificity Testing, and RT-PCR Optimization

The *Colletotrichum* GenBank accessions were downloaded from as many species and genes as possible, using multiple search strategies. In Geneious 2022.2.2 (Biomatters, Inc., Boston, MA, USA), the accessions were aligned, and duplicate sequences within species were removed, such that only unique accessions remained (Table S1). Accessions from the following 14 gene regions were visually examined for areas of high DNA polymorphism among species: ACT, ApMat, APN2, CAL, CHS, CYTB, GADPH, GS, HIS, ITS, ladA, rps3, SOD2, and TUB2. Primers and probes were then designed by eye within these areas. Primer and probe sequences were also assessed using a Nucleotide BLAST search (https://blast.ncbi.nlm.nih.gov/Blast.cgi, accessed on 1 October 2022, 10 July 2023, and 5 October 2023).

Following the initial traditional PCR testing of primer sets, the primer–probe sets were tested and optimized, using annealing temperature and primer and probe concentrations, for hydrolysis probe real-time PCR on a Bio-Rad CFX96 Connect Real-Time System. The final real-time PCR volumes were 10 µL, using the SensiFAST Probe No-ROX (Bioline, London, UK), final primer (IDT, Coralville, IA, USA) and TaqMan probe (Applied Biosystems, Waltham, MA, USA) concentrations as listed in Table 2, and 1 µL DNA (1–50 ng/µL). Cycling conditions were an initial denaturation of 95 °C for 3 minutes, followed by 40 cycles of 95 °C for 5 seconds and the optimized annealing temperature (see Table 2) for 50 seconds. Multiplex PCRs were evaluated.

Specificity was validated using 88 isolates (Table 1), including fungi from other genera, as well as apples and grapes. Hydrolysis probe real-time PCR assays were performed during three independent experiments, with three technical replicates and no-template negative controls. To assess sensitivity, standard curves for each primer–probe set were constructed, and limits of detection (LoD) were determined in 8-step dilutions (1, 0.1, 0.05, 0.01, 0.005, 0.001, 0.0005, and 0.0001 ng/µL) with 3 technical replicates, and each assay was performed three times; LoD was the lowest DNA concentration detectable across all three replicates in all three assays. Selectivity was examined by adding both *Colletotrichum* DNA (at concentrations 1, 0.1, 0.05, 0.01, 0.005, 0.001, and 0.0005 ng/µL) and apple DNA (1 ng/µL) for each assay. A two-tailed, paired Student’s t-test was used to compare Cq values, without apple DNA and with apple DNA, with significance at *p* < 0.05.

All templates that did not amplify for one of the newly designed primer–probe sets were tested with ITS1-F/ITS4 (*Colletotrichum*, *Neonectria* [53,54], Dc_09_F/Dc_09_R (*Marssonina* [55]) or COX-F/COX-R primers (plants [30]) to confirm that the DNA had no PCR inhibitors.

## 3. Results

Alignments of 1487 *Colletotrichum* GenBank accessions across 14 gene regions were visually assessed for high DNA polymorphism. Primer sets with good matches were discerned and tested for the following nine species in seven genes: *C. chrysophilum* (APN2 and ladA), *C. fioriniae* (CAL and GAPDH), *C. fructicola* (APN2 and ladA), *C. gloeosporioides* s.s. (GAPDH), *C. henanense* (ApMat), *C. noveboracense* (ApMat and ladA), *C. nymphaeae* (ACT and GAPDH), *C. siamense* (ApMat), and *C. theobromicola* (TUB2) (Table 2). After initial PCR testing, probes were designed for nine species in five genes (Table 2).

Primer sets were initially tested at 60 °C and 65 °C with traditional PCR. Sets for *C*. *chrysophilum* (CHLAD), C. *fructicola* (FRLAD), and *C*. *siamense* (SIAP) were also tested at 70 °C; additionally, CHLAD and FRLAD were tested at 74 °C. Results from these PCRs showed that an annealing temperature of at least 65 °C would be required for species-specific amplification. Therefore, for real-time PCR, testing began with an annealing temperature of 65 °C and was increased as needed (see Table S2 for the highest annealing temperatures at which the non-target species were amplified). Primer–probe concentrations were tested at final concentrations of 300 nM primers and 100 nM probe, and 600 nM primers and 200 nM probe.

Standard curve and LoD results are shown in Table 3. The real-time PCRs had high efficiencies and an LoD at 0.5 pg, except the primer–probe sets for *C. noveboracense* (NOLAD, 1 pg), and for *C. fructicola* and *C. theobromicola* (FRLAD and THTUB, 5 pg) (Table 3). The following four primer–probe set pairs were able to be duplexed: *C. fioriniae* (FICAL) and the *C. nymphaeae* (NYMG) primer–probe set, FRLAD and the *C. siamense* (SIAP) set, the *C. gloeosporioides* s.s. (GLG) set and NOLAD, and the *C. henanense* (HEAP) primer–probe set and THTUB (Table 2). Addition of apple DNA to each assay had no significant effect (Table S3).

NYMG was mostly species-specific, where *C. lupini* and a few *C. fioriniae* were amplified (Table 4). NOLAD also amplified a couple of *C. nymphaeae* (Table 4). The *C. chrysophilum* primer–probe set (CHLAD) amplified a few *C. fioriniae*, about a third of the *C. fructicola* isolates, and *C. theobromicola* (Table 4). FRLAD amplified a third of *C. chrysophilum*. Although non-specific amplifications did occur, their quantification (Cq) values were high and relative fluorescence units (RFUs) were low, as follows: Cq > 34 and RFU < 170 for CHLAD, and Cq > 37 and RFU < 100 for FRLAD, NOLAD, and NYMG (Figure S1). The primer–probe sets FICAL, GLG, HEAP, SIAP, and THTUB were species-specific, amplifying only the target species (Table 4). None amplified other fungi, apple, or grape.

In silico screening indicated that the following primer–probe sets may amplify other non-target species: *C. aenigma*, *C. camelliae*, and *C. viniferum* with FRLAD; *C. nupharicola* with NOLAD; *C. scovillei* with NYMG; *C. aeschynomenes* and *C. salsolae* with SIAP; and *C. grevilleae* and *C. grossum* with THTUB (Figure S2).

## 4. Discussion

Here, we present hydrolysis probe real-time PCR assays for the detection and identification of the following nine *Colletotrichum* species responsible for bitter rot of apple in the Mid-Atlantic U.S.A.: *C. chrysophilum*, *C. fioriniae*, *C. fructicola*, *C. gloeosporioides* s.s., *C. henanense*, *C. noveboracense*, *C. nymphaeae*, *C. siamense*, and *C. theobromicola*. After visually assessing 14 gene regions, we designed primers and probes in the following 5 gene regions for these nine species: ApMAT (*C*. *henanense*, *C*. *siamense*), CAL (*C*. *fioriniae*), GAPDH (*C*. *gloeosporioides* s.s., *C*. *nymphaeae*), ladA (*C*. *chrysophilum*, *C*. *fructicola*, *C*. *noveboracense*), and TUB2 (*C*. *theobromicola*). All were detectable from as low as 5 pg DNA, with most as low as 0.5 pg. The following four pairs of assays can be duplexed, which allows for quicker results if the whole panel is run: *C*. *fioriniae* with *C*. *nymphaeae* (both in CASC), *C*. *fructicola* with *C*. *siamense*, *C*. *gloeosporioides* with C. *noveboracense*, and *C*. *henanese* with *C*. *theobromicola* (in CGSC). These assays will provide faster identification of species than MLST, which is currently the most reliable molecular assay for species identification [22,56,57]. This is the first report of species-specific assays for *C. chrysophilum*, *C. fioriniae*, *C. henanense*, and *C. noveboracense*.

Many *Colletotrichum* species are very closely related, making any type of species delineation or identification challenging. Culture-based methods are time-intensive, require expertise, and are not always reliable [58]. MLST often requires 5–8 genes and high expertise to reliably resolve phylogenetic relationships [9,56,58,59,60]. Our primer–probe sets required as many as 20 mismatches among both the primers and probe (Figure S2) and annealing temperatures that were mainly > 68 °C in order to eliminate non-specific amplification (Table 2), underscoring the necessity of our manual, meticulous, wide-ranging search for polymorphic areas in genes from as many GenBank Accessions as we could find (Table S1).

However, the real-time PCR assays that amplified non-target species are not worrisome for us because the Cq values were high and RFUs were low for the non-target amplifications. Moreover, for most, we had another real-time PCR to confirm species identity (e.g., for any *C. fructicola* individuals that weakly amplify for CHLAD, it will strongly amplify for FRLAD).

The utility of species-specific quantitative detection assays for the *Colletotrichum* species infecting apples are numerous and far-reaching. More studies quantifying the seasonal spore release of different *Colletotrichum* spp. are needed to elucidate the key differences in the biology, ecology, epidemiology, and management of these pathogens. *Colletotrichum* management starts with cultural practices such as good orchard sanitation, as follows: removal of infection sources like diseased fruit mummies, cankered branches, and alternate hosts, and good tree canopy management for faster drying and better fungicide coverage [61,62,63]. However, quantification of propagules for different *Colletotrichum* species in various infection sources, pointing to their relative importance during the growing season, has not been explored. For example, apple buds have been largely overlooked as infection sources. The few existing reports showed that *C. acutatum* was isolated from 1.3% of apple buds in Norway [64], and 30 to 80% of apple buds in New Zealand [65], although these studies were likely dealing with several *Colletotrichum* spp. In Japan, Nekoduka et al. [61] reported fruit scars as the key overwintering sources for *Colletotrichum*. Buds are also sites for inoculum overwintering in plants such as sweet and sour cherry [66,67] and blueberry [68,69]. Even at low infection incidence, buds could play a large role as overwintering sites for *Colletotrichum* spp. [70]. Therefore, real-time PCR assays for *Colletotrichum* species will help reveal how these species survive in multiple locations in tree canopy, not being limited to cankers and mummies.

Sensitive detection assays could be used to determine the time of the first biotrophic infections in the season on apple fruit surfaces, which is the single most important event for apple producers. Knowing the time of first infections allows fungicide application against bitter rot before or during such an event, and this will mark the beginning of an effective spray program that must last until harvest. So far, the tree fruit pathologists in the main apple-growing regions of the East Coast U.S.A. have relied on observation and accumulated years of experience to recommend fungicides before the start of heavy bitter rot infection pressure. For example, in New York, the use of effective fungicides for bitter rot must not be delayed beyond 10 July, while in Pennsylvania, this cut-off period is mid to late June, and in Virginia, it is the end of May, early June. Even with the latest advances in our understanding of the ecology, epidemiology, and management of *C. fioriniae* [42,71], the more exact times of the year for the first *Colletotrichum* infections on fruit for each apple region remain undetermined.

The *Colletotrichum* species differ in their susceptibility to fungicides [10,43,44]. Our assays for rapid detection and identification of *Colletotrichum* species are critical to apple producers for refining the selection of fungicides in their spray programs. In addition to controlling bitter rot effectively, this also helps reduce the risk of *Colletotrichum* developing resistance to the single-site fungicides that growers currently rely heavily on (e.g., quinone outside inhibitors). Our detection assays can assist growers in improving fungicide programs during the growing season and in cold storage by strategically alternating classes of fungicides with different modes of action for higher control efficacy and fungicide resistance risk reduction. Once the *Colletotrichum* species is/are identified as apple rot cause, the current year spray programs can be actively improved, storage fungicides can be selected to mitigate rot spread in bins or packing lines, or fungicide choices and application strategies can be modified to prevent losses in current and the following season(s), respectively. In addition, our rapid detection and identification assays for *Colletotrichum* spp. will allow for the evaluation of different ways to improve the efficacy of existing control options for bitter rot and assist in the development of new ones.

## Figures and Tables

**Table 1:** Isolates used to test primer–probe sets.

	Taxon	Sample ID	Isolate	Host	Locality
*Colletotrichum acutatum* species complex					
	*C*. *acutatum* s.s.	VT1108	PJ51	*Lycopersicon esculentum* tomato	Auckland, New Zealand [41]
	*C*. *fioriniae*	VA-16	VA-16	Ginger Gold apple	Frederick Co. VA [22]
		VA-44	VA-44	Honeycrisp apple	Madison Co. VA [22]
		VA-53	VA-53	Honeycrisp apple	Madison Co. VA [22]
		VA-1-6	VA-1-6	Wolf River apple	Berkeley Co. WV [22]
		VA-1-16	VA-1-16	pear	[22]
		VA-1-20	VA-1-20	Ginger Gold apple	Frederick Co. VA [22]
		VA-1-66	VA-1-66	Ambrosia apple	Rappahannock Co. VA [22]
		VA-1-99	VA-1-99	Honeycrisp apple	Rappahannock Co. VA [22]
		VA-2-9	VA-2-9	Gold Rush apple	Frederick Co. VA [22]
		VA-3-59	VA-3-59	Smokehouse or Rambo apple	Fauquier Co. VA [22]
		VA-3-75	VA-3-75	Yellow York apple	Bedford Co. VA [22]
		VA-3-96	VA-3-96	Golden Delicious apple	Bedford Co. VA [22]
		VA-4-32	VA-4-32	York apple	Bedford Co. VA [22]
		VA-4-99	VA-4-99	Golden Delicious apple	Rappahannock Co. VA [22]
		VT0787	VT0787		[22]
	*C*. *godetiae*	VT1111	JA8	*Prunus dulcis* almond	CA [40]
		VT1112	S1	*Rhododendron* sp.	Helsingborg, Sweden [48]
	*C*. *johnstonii*	VT1114	PJ49	*Citrus* sp.	Clifton, New Zealand [41]
		VT1115	PJ50	*Citrus* sp.	Clifton, New Zealand [41]
	*C*. *lupini*	VT1118	PJ62	*Lupinus mutabilis*	France [47]
		VT1119	PJ64	*Lupinus alba*	Canada [45]
	*C*. *nymphaeae*	VA-1-22	VA-1-22	Ginger Gold apple	Frederick Co. VA [22]
		VA-1-24	VA-1-24	Ginger Gold apple	Frederick Co. VA [22]
		VT1124	FREC138	*Robinia pseudoacacia*	Adams Co. PA [43]
		VT1125	HC646	Honeycrisp apple	Bourbon Co. KY [44]
		VT1126	Rdl96	Empire apple	Berks Co. PA [42]
	*C*. *pyricola*	VT1127	PJ12		New Zealand [46]
	*C*. *salicis*	VT1128	FREC145	*Salix nigra* black willow	Adams Co. PA [42]
		VT1129	FREC146	*Salix nigra* black willow	Adams Co. PA [42]
*Colletotrichum gloeosporioides* species complex					
	*C*. *chrysophilum*	VA-77	VA-77	Granny Smith apple	Madison Co. VA [22]
		VA-1-83	VA-1-83	Idared apple	Frederick Co VA [22]
		VA-2-25	VA-2-25		Rappahannock Co. VA [22]
		VA-2-32	VA-2-32		Rappahannock Co. VA [22]
		VA-2-37	VA-2-37	Golden Delicious apple	Albemarle Co. VA [22]
		VA-2-67	VA-2-67	Law Rome apple	Albemarle Co. VA [22]
		VA-2-85	VA-2-85		Rappahannock Co. VA [22]
		VA-2-100	VA-2-100		Rappahannock Co. VA [22]
		VA-3-4	VA-3-4	Golden Delicious apple	Frederick Co. VA [22]
		VA-3-33	VA-3-33		Frederick Co. VA [22]
		VA-4-86	VA-4-86	Greening apple	Fauquier Co. VA [22]
		VA-5-13	VA-5-13	Granny Smith apple	Fauquier Co. VA [22]
		VA-6-19	VA-6-19	Winter Banana apple	Frederick Co. VA [22]
	*C*. *fructicola*	VA-1-32	VA-1-32	Red Delicious apple	Albemarle Co. VA [22]
		VA-1-44	VA-1-44	Golden Delicious apple	Albemarle Co. VA [22]
		VA-1-49	VA-1-49	Granny Smith apple	Nelson Co. VA [22]
		VA-1-58	VA-1-58	Golden Delicious apple	Nelson Co. VA [22]
		VA-1-68	VA-1-68	Red Delicious apple	Albemarle Co. VA [22]
		VA-1-71	VA-1-71	Golden Delicious apple	Nelson Co. VA [22]
		VA-1-78	VA-1-78	Granny Smith apple	Nelson Co. VA [22]
		VA-1-79	VA-1-79	Golden Delicious apple	Albemarle Co. VA [22]
		VA-1-90	VA-1-90	Honeycrisp apple	Nelson Co. VA [22]
		VA-1-91	VA-1-91	Granny Smith apple	Nelson Co. VA [22]
		VA-2-21	VA-2-21	Golden Delicious apple	Albemarle Co. VA [22]
		VA-2-35	VA-2-35	Golden Delicious apple	Albemarle Co. VA [22]
		VA-2-54	VA-2-54		Rappahannock Co. VA [22]
		VA-3-39	VA-3-39	Harrison apple	Albemarle Co. VA [22]
		VA-3-44	VA-3-44	Gala Supreme apple	Frederick Co. VA [22]
		VA-3-52	VA-3-52	Yates apple	Albemarle Co. VA [22]
		VA-3-54	VA-3-54	Winter White Pearmain apple	Albemarle Co. VA [22]
		VA-3-73	VA-3-73	Gala Supreme apple	Frederick Co. VA [22]
		VA-3-87	VA-3-87	Bramtot apple	Albemarle Co. VA [22]
		VA-4-12	VA-4-12	Golden Delicious apple	Fauquier Co. VA [22]
		VA-4-41	VA-4-41	GoldRush apple	Bedford Co. VA [22]
		VA-4-53	VA-4-53	Winesap apple	Albemarle Co. VA [22]
		VA-5-86	VA-5-86	Red Delicious apple	Fauquier Co. VA [22]
		VA-5-88	VA-5-88	Royal Gala apple	Frederick Co. VA [22]
		VA-6-15	VA-6-15	Royal Gala apple	Frederick Co. VA [22]
		VA-6-16	VA-6-16	Buckeye Gala apple	Botetourt Co. VA [22]
		VA-6-28	VA-6-28	Pink Lady apple	Albemarle Co. VA [22]
		VA-6-56	VA-6-56	Buckeye Gala apple	Botetourt Co. VA [22]
		VA-6-59	VA-6-59	Buckeye Gala apple	Botetourt Co. VA [22]
		VT1109	HC540	Honeycrisp apple	Bourbon Co. KY [44]
	*C*. *gloeosporioides* s.s.	VT1104	DLC8	apple	Frederick Co. MD [43]
	*C*. *henanense*	VT1105	SHB6	apple	Westmoreland Co. PA [45]
		VT1113	SHB5a	apple	Westmoreland Co. PA [43]
	*C*. *kahawae* clade	VT1116	HC278	*Malus pumila*	KY [3]
		VT1117	HC292	*Malus pumila*	KY [3]
	*C*. *noveboracense*	VT1106	AFKH109	Idared apple	Columbia Co. NY [44]
		VT1120	PMBrms-1	apple	Adams Co. PA [44]
		VT1121	PMCMS-6751	apple	Lehigh Co. PA [44]
		VT1122	Coll940	*Juglans nigra*	Cherokee Co. OK [44]
		VT1123	PMEssl-10a	apple	Lycoming Co. PA [44]
	*C*. *siamense*	VA-6-10	VA-6-10	Granny Smith apple	Amherst Co. VA [22]
		VT1130	DLC6a	apple	Frederick Co. MD [43]
		VT1131	KY146	apple	Clinton Co. KY [44]
		VT1132	KY8	apple	Harlan Co. KY [44]
	*C*. *theobromicola*	VA-41	VA-41	Granny Smith apple	Nelson Co. VA [22]
Other fungi					
	*Botryosphaeria dothidea*	VT0745	VT0745	grape	Frederick Co. VA
	*Diaporthe* sp.	VT0748	VT0748	grape	Frederick Co. VA
	*Diplocarpon coronariae*	VT1136	BMO8	apple	Adams Co. PA [50]
		VT1137	BMO9	apple	Adams Co. PA [50]
		VT1138	Vtech4	apple	Frederick Co. VA [50]
		VT1139	Vtech5	apple	Frederick Co. VA [50]
	*Erysiphe necator*	VT0688	VT0688	grape	Frederick Co. VA
	*Monilinia fructicola*	VT1110	Mfa1	Jonamac apple	Lancaster Co. PA [51,52]
	*Neonectria ditissima*	VT1133	EUC1-T-1	apple	Floyd Co. VA
	*Penicillium expansum*	VT1135	TDL12.1	—	[49]
	*Pestalotiopsis maculans*	VT0746	VT0746	grape	Frederick Co. VA
	*Phomopsis viticola*	VT0005	VT0005	*Vitis*	Frederick Co. VA
	*Plasmopara viticola*	VT0693	VT0693	*Vitis*	Shenandoah Co. VA
Plants					
	*Malus domestica* McIntosh	VT0695	—	—	Frederick Co. VA
	*Vitis vinifera*	GRAPE DNA	—	—	Frederick Co. VA

**Table 2:** Primer and probe target species and gene region, name, sequence and fluorophore, final concentration, anneal temperature, and amplicon size.

Species	Gene Region	Primer and Probe	Sequence (5′-3′)	Final Concentration (nM)	Anneal T (°C)	Amplicon Size (bp)
*C*. *chrysophilum*	ladA	CHLADF2	CAT CGT GGC TGT AAT TTT GGA TGT TTC	300	72	164
		CHLADR	CTT GCC GAA TCC TTC GCT GGT GGT CAC GGC CGA T	300		
		CHLADP	6FAM-GAC ACC AGT CGC CTT GAC GTG G-MGBNFQ	100		
*C*. *fioriniae*	calmodulin	FICALF	TTT ACG CAG CAA CCA CTG GCA ACC ATC	600	69	182
		FICALR	GTC TCT GAT TAG CAC TAT CTA CAT GC	600		
		FICALP	VIC-TTC AAG GTG AGA AGA TCT GGC GCA A-MGBNFQ	200		
*C*. *fructicola*	ladA	FRLADF2	TCT CAT GAC AGG AGC TTC CGA GAT TTC	600	70	164
		FRLADR	GCT GCC GAA CCC CTC ATT GGT GGT CAC GGC CGA C	600		
		FRLADP	VIC-AAC ACC AGT CGC CTT AAC GTG A-MGBNFQ	200		
*C*. *gloeosporioides* s.s.	GAPDH	GLGF	CTC CAA GCT CGW CAT GAC TTC AC	600	68	114
		GLGR	GAT TTC AAT TGG CAT TAA TTC ATR ATG GCC	600		
		GLGP	6FAM-GCC GCC CGC GTT TAG TAC AC-MGBNFQ	200		
*C*. *henanense*	ApMat	HEAPF	TGA CTT GGT CAT CGA TTC GCT TCC CG	300	65	141
		HEAPR	GCG AGG ATG GTT CTC GAT TCG	300		
		HEAPP	VIC-CCT TGC GCC AGA AAC CAA CCC ACC T-MGBNFQ	100		
*C*. *noveboracense*	ladA	NOLADF	GGG AAG TAT AGT CAG CGC ATT G	300	68	357
		NOLADR	TAA TCG CCG TCT CTC GTT CGT TCG AC	300		
		NOLADP	VIC-CGT CAT GAC TGG AAT TTG TGA TGT TCC-MGBNFQ	100		
*C*. *nymphaeae*	GAPDH	NYMGF	GAT AAC ACC AGC TTC GTC GAT ATC	300	69	132
		NYMGR	TCT GTC AGC AAG TTT TGT CTC GGC	300		
		NYMGP	6FAM-GAT TGG GCT TGT TGT AAC GAC ACG-MGBNFQ	100		
*C*. *siamense*	ApMat	SIAPF	ACT GAT ATC GGC GCT GCC AG	300	70	168
		SIAPR	GAA GGG AAT CGA TGG CCA GAT GTG	300		
		SIAPP	6FAM-CGA CCT AAG GTT GTC TTT GTG TCC TAG-MGBNFQ	100		
*C*. *theobromicola*	beta-tubulin	THTUBF	CTT TCA CCC GAG TTC CAT GTT CAC C	600	65	181
		THTUBR	GCG AGA GAT TAG CCC TTA GCC CTG C	600		
		THTUBP	6FAM-CGT CAA TCC GAC CCC CTA CTG CG-MGBNFQ	200		
Other primer or primer–probe sets tested but produced too much non-specific amplification:						
*C*. *chrysophilum*	APN2	CHAPNF2	GGC AAT CTA CAC CCG CAA CGC G	300	72	131
		CHAPNR	GGT ACC CGC CGA TAT GCT G	300		
		CHAPNP	VIC-CGT GGC GCG ACC TGC CCC CG-MGBNFQ	100		
*C*. *fioriniae*	GAPDH	FIGF	TAC AAT AAC ACC AGC TTC ATC GGT AAC	100	65	154
		FIGR	TCT GTC AGC AAA TTT TGT TTG GGC	100		
*C*. *fructicola*	APN2	FRAPNF	GGC AAT CTA CAC CCG CAA CGC A	100	65	131
		FRAPNR	GGT ACC CGC CGA TGT GCT G	100		
*C*. *noveboracense*	ApMat	NOAPF	GTG AGG ACC ATT GAT TTG CCC ACA TGT T	100	65	116
		NOAPR	GGA TCA GAC CTA GCT ATT CCC GTG ATG	100		
*C*. *nymphaeae*	ACT	NYACTF	CGC AGA CCG CAA TCT TCT CCG TCA GG	100	65	150
		NYACTR	GCA GGA GAT GGC ATT GCC GCA GC	100		

**Table 3:** Efficiency (E), R^2^, slope, y-intercept, Cq at 1000 pg, and limit of detection (LoD) of primer–probe sets.

Primer–Probe Set (*Colletotrichum* Species, Gene)	E	R^2^	slope	y-Intercept	Cq at 1000 pg	LoD in pg (Cq)
CHLAD (*C*. *chrysophilum*, ladA)	94.6%	0.992	−3.546	22.525	23	0.5 (36)
FICAL (*C*. *fioriniae*, calmodulin)	92.1%	0.987	−3.630	22.867	23	0.5 (36)
FRLAD (*C. fructicola*, ladA)	99.5%	0.963	−3.346	28.457	29	5 (36)
GLG (*C*. *gloeosporioides* s.s., GAPDH)	108.2%	0.978	−3.158	22.104	22	0.5 (33)
HEAP (*C*. *henanense*, ApMat)	92.1%	0.991	−3.529	22.851	23	0.5 (35)
NOLAD (*C*. *noveboracense*, ladA)	90.5%	0.991	−3.529	22.9851	24	1 (35)
NYMG (*C*. *nymphaeae*, GAPDH)	92.6%	0.991	−3.529	22.851	23	0.5 (35)
SIAP (*C*. *siamense*, ApMat)	93.2%	0.983	−3.713	22.752	22	0.5 (35)
THTUB (*C*. *theobromicola*, beta-tubulin)	101.3%	0.921	−3.226	27.215	27	5 (35)

**Table 4:** Number of isolates per species that were amplified for each primer–probe set (*n* = total number of isolates, number amplified/number tested). Amplifications are in bold. Primer–probe sets: CHLAD is for *C*. *chrysophilum* in gene ladA, FICAL *C*. *fioriniae* in calmodulin, FRLAD *C*. *fructicola* in ladA, GLG *C*. *gloeosporioides* s.s. in GAPDH, HEAP *C*. *henanense* in ApMat, NOLAD *C*. *noveboracense* in ladA, NYMG *C*. *nymphaeae* in GAPDH, SIAP *C*. *siamense* in ApMat, and THTUB *C*. *theobromicola* in beta-tubulin.

	Taxon	CHLAD	FICAL	FRLAD	GLG	HEAP	NOLAD	NYMG	SIAP	THTUB
*Colletotrichum acutatum* species complex										
	*C*. *acutatum* s.s. (n = 1)	0/1	0/1	0/1	0/1	0/1	0/1	0/1	0/1	0/1
	*C*. *fioriniae* (n = 15)	**3/14**	**13/15**	0/14	0/15	0/15	0/14	**2/15**	0/15	0/15
	*C*. *godetiae* (n = 2)	0/2	0/2	0/2	0/2	0/2	0/2	0/2	0/2	0/2
	*C*. *johnstonii* (n = 2)	0/2	0/2	0/2	0/2	0/2	0/2	0/2	0/2	0/2
	*C*. *lupini* (n = 2)	0/2	0/2	0/2	0/2	0/2	0/2	**2/2**	0/2	0/2
	*C*. *nymphaeae* (n = 5)	0/5	0/5	0/5	0/5	0/5	**2/5**	**5/5**	0/5	0/5
	*C*. *pyricola* (n = 1)	0/1	0/1	0/1	0/1	0/1	0/1	0/1	0/1	0/1
	*C*. *salicis* (n = 2)	0/2	0/2	0/2	0/2	0/2	0/2	0/2	0/2	0/2
*Colletotrichum gloeosporioides* species complex										
	*C*. *chrysophilum* (n = 13)	**13/13**	0/13	**4/13**	0/13	0/13	0/13	0/13	0/13	0/13
	*C*. *fructicola* (n = 30)	**8/30**	0/30	**30/30**	0/30	0/30	0/30	0/30	0/30	0/30
	*C*. *gloeosporioides* s.s. (n = 1)	0/1	0/1	0/1	**1/1**	0/1	0/1	0/1	0/1	0/1
	*C*. *henanense* (n = 2)	0/2	0/2	0/2	0/2	**2/2**	0/2	0/2	0/2	0/2
	*C*. *kahawae* clade (n = 2)	0/2	0/2	0/2	0/2	0/2	0/2	0/2	0/2	0/2
	*C*. *noveboracense* (n = 5)	0/5	0/5	0/5	0/5	0/5	**5/5**	0/5	0/5	0/5
	*C*. *siamense* (n = 4)	0/4	0/4	0/4	0/4	0/4	0/4	**1/4**	**4/4**	0/4
	*C*. *theobromicola* (n = 1)	**1/1**	0/1	0/1	0/1	0/1	0/1	0/1	0/1	**1/1**
Other fungi										
	*Botryosphaeria dothidea* (n = 1)	0/1	0/1	0/1	0/1	0/1	0/1	0/1	0/1	0/1
	*Diaporthe* sp. (n = 1)	0/1	0/1	0/1	0/1	0/1	0/1	0/1	0/1	0/1
	*Diplocarpon coronariae* (n = 4)	0/4	0/4	0/4	0/3	0/4	0/4	0/4	0/4	0/4
	*Erysiphe necator* (n = 1)	0/1	0/1	0/1	0/1	0/1	0/1	0/1	0/1	0/1
	*Monilinia fructicola* (n = 1)	0/1	0/1	0/1	0/1	0/1	0/1	0/1	0/1	0/1
	*Neonectria ditissima* (n = 1)	0/1	0/1	0/1	0/1	0/1	0/1	0/1	0/1	0/1
	*Penicillium expansum* (n = 1)	0/1	0/1	0/1	0/1	0/1	0/1	0/1	0/1	0/1
	*Pestalotiopsis maculans* (n = 1)	0/1	0/1	0/1	0/1	0/1	0/1	0/1	0/1	0/1
	*Phomopsis viticola* (n = 1)	0/1	0/1	0/1	0/1	0/1	0/1	0/1	0/1	0/1
	*Plasmopara viticola* (n = 1)	0/1	0/1	0/1	0/1	0/1	0/1	0/1	0/1	0/1
Plants										
	*Malus domestica* (n = 1)	0/1	0/1	0/1	0/1	0/1	0/1	0/1	0/1	0/1
	*Vitis vinifera* (n = 1)	0/1	0/1	0/1	0/1	0/1	0/1	0/1	0/1	0/1

## Data Availability

The raw data supporting the conclusions of this article will be made available by the authors on request.

## Supplementary Materials

The following supporting information can be downloaded at: https://www.mdpi.com/article/10.3390/microorganisms12050878/s1, Figure S1: Amplification plots showing high Cq values and low RFU values of non-specific amplifications; Figure S2: Alignments of available *Colletotrichum* accessions at primer and probe sites for each primer–probe set; Table S1: List of GenBank accessions used to assess areas of DNA polymorphism among *Colletotrichum* spp. (n = 1487); Table S2: Highest annealing temperature (°C) at which target and non-target species amplified for each primer–probe set; Table S3: Real-time PCR standard curve Cq values and effect of apple DNA on Cq (NA = no amplification during assay with apple DNA, nt = not tested because it was below LoD).
