# Supplementary material for: New Species-Specific Real-Time PCR Assays for Colletotrichum Species Causing Bitter Rot of Apple
Source: Microorganisms. 2024 Apr 27;12(5):878. doi: 10.3390/microorganisms12050878 (PMC11123832; doi:10.3390/microorganisms12050878)
Supplement: Supplementary file 1 [file microorganisms-12-00878-s001.zip › microorganisms-2916949-supplementary.pdf]

Table S1. List of GenBank accessions used to assess areas of DNA polymorphism among *Colletotrichum* spp. (n=1,487). Available as a Microsoft Office Excel spreadsheet.

Table S2. Highest annealing temperature (°C) at which target and non-target species amplified for each primer-probe set (— = never amplified at any temperature). Target amplifications are in **bold**.

Taxon	CHLAD	FICAL	FRLAD	GLG	HEAP	NOLAD	NYMG	SIAP	THTUB
Colletotrichum acutatum species complex									
C. acutatum s.s.	—	60	—	—	—	—	—	—	—
C. fioriniae	72	**69**	—	—	—	—	69	—	—
C. godetiae	—	—	—	—	—	—	—	—	—
C. johnstonii	—	—	—	—	—	—	—	—	—
C. lupini	—	—	—	—	—	—	69	—	—
C. nymphaeae	—	67	—	—	—	68	**69**	—	—
C. pyricola	—	—	—	—	—	—	—	—	—
C. salicis	—	—	—	—	—	—	—	—	—
Colletotrichum gloeosporioides species complex									
C. chrysophilum	**72**	67	70	65	—	—	—	—	—
C. fructicola	72	—	**70**	—	—	—	—	—	—
C. gloeosporioides s.s.	68	67	68	**68**	—	—	—	—	—
C. henanense	70	—	68	—	**65**	—	—	—	—
C. kahawae clade	68	—	—	—	—	—	—	—	—
C. noveboracense	—	—	—	67	—	**68**	—	—	—
C. siamense	68	—	68	67	—	—	69	**70**	—
C. theobromicola	72	—	—	—	—	—	—	—	**65**
Other fungi									
Botryosphaeria dothidea	—	—	—	—	—	—	—	—	—
Diaporthe sp.	—	67	—	—	—	—	—	—	—
Diplocarpon coronariae	—	—	—	—	—	—	—	—	—
Erysiphe necator	—	—	—	—	—	—	—	—	—
Neonectria ditissima	—	—	—	—	—	—	—	—	—
Penicillium expansum	—	—	—	—	—	—	—	—	—
Pestalotiopsis maculans	—	—	—	—	—	—	—	—	—
Phomopsis viticola	—	—	—	—	—	—	—	—	—
Plasmopara viticola	—	—	—	—	—	—	—	—	—
Plants									
Malus domestica	—	—	—	—	—	—	—	—	—
Vitis vinifera	—	—	—	—	—	—	—	—	—

Table S3. Real-time PCR standard curve Cq values and effect of apple DNA on Cq (NA = no amplification during assay with apple DNA, nt = not tested because it was below LoD).

Species	Primer-probe set	[DNA] (ng/µL)	Cq mean ± SD	Ratio fungal DNA: apple DNA	Cq mean ± SD
C. chrysophilum	CHLAD	1	24.09 ± 0.04	1:1	22.92 ± 2.48
		0.1	27.44 ± 0.16	1:10	28.81 ± 1.54
		0.05	28.35 ± 0.14	1:20	29.31 ± 1.01
		0.01	31.07 ± 0.03	1:100	32.74 ± 0.69
		0.005	32.52 ± 0.12	1:200	32.28 ± 0.65
		0.001	34.77 ± 0.49	1:1,000	38.14 ± 0.45
		0.0005	37.03 ± 1.21	1:2,000	NA
					P = 0.18299
C. fioriniae	FICAL	1	25.75 ± 0.15	1:1	23.51 ± 0.15
		0.1	28.95 ± 0.11	1:10	27.46 ± 0.14
		0.05	29.99 ± 0.10	1:20	29.20 ± 0.35
		0.01	32.28 ± 0.37	1:100	32.15 ± 0.63
		0.005	34.00 ± 0.22	1:200	37.46 ± 0.27
		0.001	35.92 ± 0.29	1:1,000	37.38 ± 0.00
		0.0005	36.85 ± 0.39	1:2,000	36.41 ± 0.00
					P = 0.82347
C. fructicola	FRLAD	1	32.31 ± 0.17	1:1	31.15 ± 0.07
		0.1	34.95 ± 0.76	1:10	34.27 ± 0.29
		0.05	36.08 ± 0.66	1:20	35.35 ± 0.21
		0.01	38.09 ± 0.74	1:100	38.37 ± 1.52
		0.005	39.32 ± 0.83	1:200	37.62 ± 0.00
		0.001	NA	1:1,000	nt
		0.0005	NA	1:2,000	nt
					P = 0.07049
C. gloeosporioides	GLG	1	22.35 ± 0.22	1:1	22.58 ± 0.20
		0.1	25.92 ± 0.18	1:10	25.65 ± 0.30
		0.05	26.45 ± 0.28	1:20	26.58 ± 0.55
		0.01	28.2 ± 0.17	1:100	29.99 ± 0.39
		0.005	29.16 ± 0.34	1:200	31.51 ± 0.70
		0.001	31.57 ± 0.55	1:1,000	NA
		0.0005	31.90 ± 0.52	1:2,000	NA
					P = 0.47416
C. henanense	HEAP	1	23.54 ± 0.14	1:1	22.83 ± 0.12
		0.1	27.06 ± 0.15	1:10	26.60 ± 0.18
		0.05	27.86 ± 0.22	1:20	27.48 ± 0.17
		0.01	30.85 ± 0.23	1:100	31.58 ± 0.26
		0.005	31.32 ± 0.14	1:200	31.32 ± 0.60
		0.001	34.40 ± 1.01	1:1,000	34.41 ± 0.52
		0.0005	34.89 ± 0.48	1:2,000	34.87 ± 0.18
					P = 0.52883

C. noveboracense	NOLAD	1	25.30 ± 0.86	1:1	25.10 ± 0.46
		0.1	29.11 ± 0.14	1:10	28.67 ± 0.25
		0.05	30.48 ± 0.17	1:20	29.86 ± 0.26
		0.01	32.64 ± 0.31	1:100	32.08 ± 0.26
		0.005	33.96 ± 0.28	1:200	33.53 ± 0.47
		0.001	36.22 ± 0.31	1:1,000	34.94 ± 0.00
		0.0005	36.73 ± 1.22	1:2,000	nt
					P = 0.05563
C. nymphaeae	NYMG	1	23.84 ± 0.31	1:1	23.58 ± 0.10
		0.1	27.03 ± 0.06	1:10	27.22 ± 0.06
		0.05	28.33 ± 0.09	1:20	28.22 ± 0.13
		0.01	30.72 ± 0.42	1:100	30.79 ± 0.27
		0.005	31.94 ± 0.34	1:200	33.16 ± 0.53
		0.001	35.40 ± 2.46	1:1,000	35.36 ± 0.45
		0.0005	36.53 ± 1.08	1:2,000	NA
					P = 0.44967
C. siamense	SIAP	1	23.72 ± 0.18	1:1	23.33 ± 0.35
		0.1	26.89 ± 0.44	1:10	26.63 ± 0.29
		0.05	27.95 ± 0.08	1:20	27.58 ± 0.24
		0.01	30.28 ± 0.19	1:100	31.11 ± 0.08
		0.005	32.08 ± 0.04	1:200	32.41 ± 0.60
		0.001	34.06 ± 0.93	1:1,000	35.60 ± 1.71
		0.0005	35.30 ± 0.81	1:2,000	35.20 ± 0.00
					P = 0.44250
C. theobromicola	THTUB	1	28.81 ± 0.17	1:1	29.35 ± 0.36
		0.1	31.78 ± 0.23	1:10	31.71 ± 0.50
		0.05	32.59 ± 0.22	1:20	33.71 ± 0.41
		0.01	36.41 ± 1.37	1:100	35.97 ± 0.75
		0.005	37.07 ± 1.36	1:200	37.44 ± 1.07
		0.001	NA	1:1,000	nt
		0.0005	NA	1:2,000	nt
					P = 0.31748

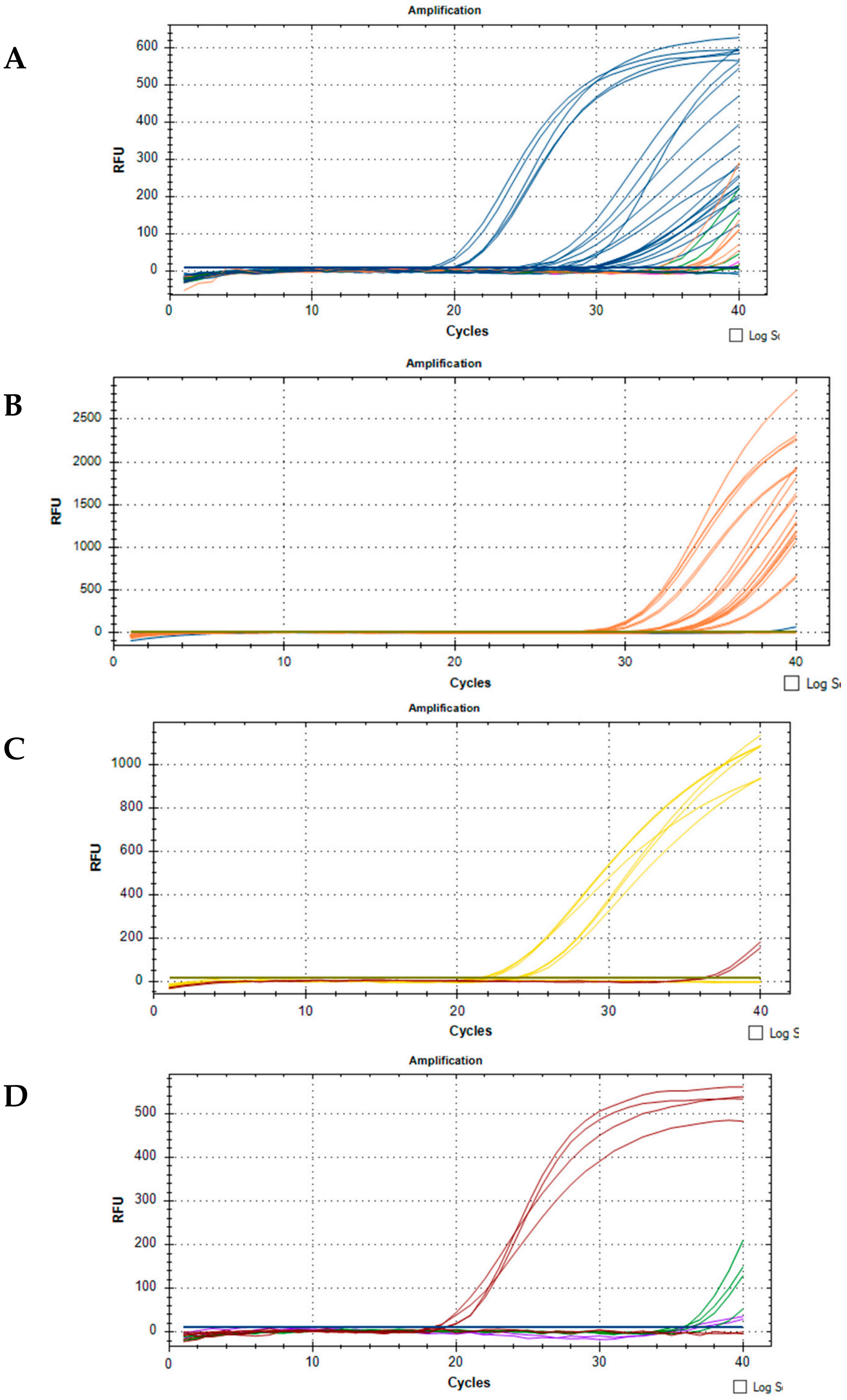

Figure S1. Amplification plots showing high Cq values and low RFU values of non-specific amplifications: A) CHLAD primer-probe set, B) FRLAD, C) NOLAD, and D) NYMG. Blue = *C. chrysophilum* (A, B), green = *C. fioriniae* (A, D), orange = *C. fructicola* (A, B), purple = *C. lupini* (D), yellow = *C. noveboracense* (C), dark red = *C. nymphaeae* (C, D), and pink = *C. theobromicola* (A). No-template controls are in the target species color.

	5′ CHLADF2 22 bp CHLADP 59 bp CHLADR 3′
C. chrysophilum	CATCGTGGCTGTAATTTTGGATGTTTC...GACACCAGTCGCCTTGACGTGG...ATCGGCCGTGACCACCAGCGAAGGATTCGGCAAG
C. aenigma	TGTCATGACTGGAGCTTCCGAGGTCTC...AACACCAGTCGCCTTGACGTGG...ATCGGCCGTGACCACCAACGAGGGGTTCGCCAGC
C. camelliae	CGTCGTGACTGGAACTTCCGAGATCTG...AACACCAGTTGCCTTGACGTGG...GTCGGCCGTGACCACCAGCGAGGGGTTCGGCAGC
C. fructicola	TCTCATGACAGGAGCTTCCGAGATTTC...AACACCAGTCGCCTTAACGTGA...GTCGGCCGTGACCACCAATGAGGGGTTCGGCAGC
C. gloeosporioides	CGTCATGACTGAAA-TTGTGGTGCTTC...GACACCAGTCGCCTTGACGTGG...GTCGGCCGTGACCACCAGTGAGGGGTTCGGCAGC
C. noveboracense	CGTCATGACTGGAATTTGTGATGTTCC...AACACCTGTCGCTTTGACGTGG...ATCGGCCGTGACCACCAACGAGGGGTTCGGCAGC
C. nupharicola	CGTCATGACTGGAATTTGTGATGTTCC...AACACCTGTCGCTTTGACGTGG...ATCGGCCGTGACCACCAACGAGGGGTTCGGCAGC
C. siamense	CGTCATGACTGAAA-TTGTGGCGTTTC...AACACCAGTCGCCTTGACGTGG...GTCGGCCGTGACCACCAGCGAAGGGTTCGGCAAG
C. theobromicola	T-ACACATCAGGAGTTTCTCTTCTCGG...AACACCAGTCGCCTTGACGTGG...GTCGGCGGTGACGACGAGCGAGGGGTTCGGCAGC
C. tropicale	CGTCATGACTGAAA-TCGTGGCGTGTC...AACACCAGTCGCCTTGACGTGG...ATCGGCCGTTACCACTAGCGAAGGGTTCGGCAAG
C. viniferum	TGTCATGACTGGAGCTTCCGAGGTCTC...AACACCAGTCGCCTTGACGTGG...ATCGGCCGTGACCACCAGTGAAGGGTTCGGCAAG

	5′ FICALF 101 bp FICALP 3 bp FICALR 3′
C. fioriniae	TTTACGCAGCAACCACTGGCAACCATC...TTCAAGGTGAGAAGATCTGGCGCAA...GCATGTAGATAGTGCTAATCAGAGAC
C. acutatum	TCTACACAACAACTACTGGCAATCACG...TTCAAGGTAAGAAGAGCTAGTGCAA...GCACGTGGATAATGCTGACCAGAGGA
C. aenigma	TGGACATGCGGAATCCTGCGT------...TTCAAGGTTGGCTACGACGGTGCTT...CGCTGCGATTGATGTTGACTCTGAGT
C. chrysophilum	TGGACACGCGGAATCCTGCGT------...TTCAAGGTTGGCTACGACGGTGCTT...CGCTGCGATTGATGTTGACTCTGAGT
C. conoides	TGGACATGCGGAATCCTGCGT------...TTCAAGGTTGGCTACGACGGTGCTT...CGCTGCGATCGATGTTGACTCTGAAT
C. fructicola	TGGACACGCGGAATCCTGCGT------...TTCAAGGTTGGCTACGACGGTGCTT...CGCTGCGATTGATGTTGACTCTGAGT
C. gloeosporioides	TGGACATGCGGAATTCTGCGC------...TTCAAGGTTGGCTATGACGGTGCTT...CGCTGCGATCGACGTTGACTCT-AAT
C. henanense	TGGACATGCGGAATCCTGCAT------...TTCAAGGTTGGCTACGGCAGTGTTT...CGCTGCGATCGGTGCTGACTCCGCAT
C. hymenocallidis	TGGACATGCGGAATCCTGCGT------...TTCAAGGTTGGCTACGACGGTGCTT...CGCTGCGATATATGTTGACTCTGAAT
C. nymphaeae	TCTACACAACAACTACTGGCAATCACG...TTCAAGGTAAGAAGAGCTAGTGCAA...GCACGTGGATAATGCTGACCAGAGGA
C. siamense	TGGACATGCGGAATCCTGCGT------...TTCAAGGTTGGCTACGACGGTGCTT...CGCTGCGATATATGTTGACTCTGAAT
C. theobromicola	TTGACATACGGAAATCCGTGT------...TTCAAGGTCAGCTACGAGAATGTTT...TGCTGCGGTCGATGTTGACTCTGAAC

	5′ FRLADF2 22 bp FRLADP 59 bp FRLADR 3′
C. fructicola	TCTCATGACAGGAGCTTCCGAGATTTC...AACACCAGTCGCCTTAACGTGA...GTCGGCCGTGACCACCAATGAGGGGTTCGGCAGC
C. aenigma	TGTCATGACTGGAGCTTCCGAGGTCTC...AACACCAGTCGCCTTGACGTGG...ATCGGCCGTGACCACCAACGAGGGGTTCGCCAGC
C. camelliae	CGTCGTGACTGGAACTTCCGAGATCTG...AACACCAGTTGCCTTGACGTGG...GTCGGCCGTGACCACCAGCGAGGGGTTCGGCAGC
C. chrysophilum	CATCGTGGCTGTAATTTTGGATGTTTC...GACACCAGTCGCCTTGACGTGG...ATCGGCCGTGACCACCAGCGAAGGATTCGGCAAG
C. gloeosporioides	CGTCATGACTGAAA-TTGTGGTGCTTC...GACACCAGTCGCCTTGACGTGG...GTCGGCCGTGACCACCAGTGAGGGGTTCGGCAGC
C. noveboracense	CGTCATGACTGGAATTTGTGATGTTCC...AACACCTGTCGCTTTGACGTGG...ATCGGCCGTGACCACCAACGAGGGGTTCGGCAGC
C. nupharicola	CGTCATGACTGGAATTTGTGATGTTCC...AACACCTGTCGCTTTGACGTGG...ATCGGCCGTGACCACCAACGAGGGGTTCGGCAGC
C. siamense	CGTCATGACTGAAA-TTGTGGCGTTTC...AACACCAGTCGCCTTGACGTGG...GTCGGCCGTGACCACCAGCGAAGGGTTCGGCAAG

C. theobromicola	T-ACACATCAGGAGTTTCTCTTCTCGG...AACACCAGTCGCCTTGACGTGG...GTCGGCGGTGACGACGAGCGAGGGGTTCGGCAGC
C. tropicale	CGTCATGACTGAAA-TCGTGGCGTGTC...AACACCAGTCGCCTTGACGTGG...ATCGGCCGTTACCACTAGCGAAGGGTTCGGCAAG
C. viniferum	TGTCATGACTGGAGCTTCCGAGGTCTC...AACACCAGTCGCCTTGACGTGG...ATCGGCCGTGACCACCAGTGAAGGGTTCGGCAAG

	5' GLGF 45 bp GLGP 5 bp GLGR 3'
C. gloeosporioides	CTCCAAGCTCGWCATGACTTCAC...GCCGCCCGCGTTTAGTACAC...GGCCATYATGAATTAATGCCAATTGAAATC
C. aenigma	CTCCAAACTCGCCATGACTTCAC...GCCACCCGCGTTTGGTAAAC...GGCAGTCATGAATGGAGGCCAATTGAAACC
C. alienum	CTCCAAACTCGCCATGACTTCAC...GCCACCCGCGTTTGGTAAAC...GGCCGTCATGAATGGAGGCCAATTGAAACC
C. aotearoa	CTCCAAACTCGTCATGACTCTCA...GCCGCCCGCATCTGGTAGAC...GGCTATCTTGACTTGATGCCAATTGAAACC
C. camelliae	CTCCAAACTCGCCATGACTTCGC...GCCACCCGCGTTTGGTAAAT...GGCCGTCATGAATGGAGGCCAATTGAAACC
C. chrysophilum	CTCCAAACTCGCCATGACTTCAC...GCCACCCGCGTTTGGTAAAC...GGCCGTCATGAATGGAGGCCAATTGAAACC
C. conoides	CTGCAAACTCGCCATGACTTCAC...GCCACCCGCGTTTGGTAAAC...GGCAGTCATGAATGGAGGCCAATTGAAACC
C. fructicola	CTCCAAACTCGCCATGACTTCAC...GCCACCCGCGTTTGGTAAAT...GGCCGTCATGAATGGAGGCCAATTGAAACC
C. henanense	CTCCAAACTCGTCATGACT-CTC...GCCGCCCGCATCTGGTAGAC...GGCTATCGTGACTTGATGCCAATTGAAACC
C. musae	CTCCAAACTCGCCATGACTTCAC...GCCACCCGCGTTTGGTAAAT...GGCCGTCATGAATGGAGGCCAATTGAAACC
C. queenslandicum	CTCCAAACTCGCCATGACTTCAC...GCCACCCGCGTTTGGTAAAC...GGCCGTCATGAATGGAGGCCAATTGAAACC
C. siamense	CTCCAAACTCGCCATGACTTCAC...GCCACCCGCGTTTGGTAAAC...GGCCGTCATGAATGGAGGCCAATTGAAACC
C. theobromicola	CTCCAAACTCGCCACTACTTCAC...CCCGCCTGTATTTGGCAGAC...GGCCAACATGAATTGATGCCAATTGATACC
C. tropicale	CTCCAAAATCGCCATGACTTCAC...GCCACCCGCGTTTGGTAAAT...GGCCGTCATGAATGGAGGCCAATTGAAACC

	5' HEAPF 19 bp HEAPP 50 bp HEAPR 3'
C. henanense	TGACTTGGTCATCGATTCGCTTCCCG...CCTTGCGCCAGAAACCAACCCACCT...CGAATCGAGAACCATCCTCGC
C. aenigma	TCGTCGATTC--------ACTTCCCG...C-CTGCGCGAGAAACCAACAGACCT...GGAACCAAGAATCATCCTGGA
C. aeschynomenes	TCATCGATTC---------TTTGCCG...CCCTGCACCAGAAAACAACAGACCT...GGAACCAAGAATCATCCTGGC
C. alatae	TCATCAGTTC--------CATTCCCG...CCCTGCGCCAGAAACCAACAGATCT...TGAATCAAGAATCATCCTGGG
C. alienum	TCGTCGATTC--------ACTTCCCG...C-CTGCGCGAGAAACCAACAGACCT...GGAACCAAGAGTCATCCTGGA
C. aotearoa	TCATCGATTC--------GCTTCCCG...CCTTGCGCCAGAAACCAACACACCT...CGAACCAAGAACCATCCTGGT
C. camelliae	TCATCGATTC--------GCTTCCCG...CCTTGCGCCAGAAACCCACAAACCT...CGAACCAAGAACCATCCTGGT
C. chrysophilum	TCGTCGATTC--------ACTTCCCG...C-CTGCGCGAGAAACCAACAGACCT...GGAACCAAGAATCATCCTGGA
C. clidemiae	TCATCGATTC--------GCTTCCCG...CCTTGCGCCAGAAACCCACAAACCT...CGAACCAAGAACCATCCTGGT
C. conoides	TCGTCGATTC--------ACTTCCCG...C-CTGCGCGAGAAACCAACAGACCT...GGAACCAAGAATCATCCTGGA
C. fructicola	TCGTCGATTC--------ACTTCCCG...C-CTGCGCGAGAAACCAACAGACCT...GGAACCAAGAATCATCCTGGA
C. gloeosporioides	TCATCGATTC--------CCTTCCTA...CCCTGCGCCGGAAACCAACAGACCT...CGAACCAAGAATCATCCTGGC
C. horii	TCGTCGATTC--------CCTTCCCG...CTCTGCGCCAGAGATCAAAAAACCT...CAAACCGAGAACCATCCTGGA
C. jiangxiense	TCATCGATTC--------GCTTCCCG...CCTTGCGCCAGAAACCCACAAACCT...CGAACCAAGAACCATCCTGGT
C. musae	TCGTCGATTC--------ACTTCCCG...C-CTGCGCGAGAAACCAACAGACCT...GGAACCAAGAATCATCCTGGA

C. noveboracense	TCGTCGATTC--------ACTTCCCG...C-CTGCGCGAGAAACCAACAGACCT...GGAACCAAGAATCATCCTGGA
C. perseae	TCGTCGATTC--------ACTTCCCG...C-CTGCGCGAGAAACCAACAGACCT...GGAACCAAGAATCATCCTGGA
C. psidii	TCATCGATTC--------GCTTCGCG...CCTTGCGCCAGAAACCCACAAACCT...CGAACCAAGAACCATCCTGGT
C. queenslandicum	TCATCGATTC--------CCTTCCCG...CCCTGCGCCAGAAACCAATAGACCT...GGACCCAAGAATCATCCTGGC
C. salsolae	CCATCGATTT--------CCTTCCCG...CCCTGCGCCAGAAACCAACAGACCT...GGAACCAAGAATCATCCTGGC
C. siamense	CCATCGATTC--------CCTTCCCG...CCCTGCGCCAGAAACCAACAGACCT...GGAACCAAGAATCATCCTGGC
C. theobromicola	CCAGCAACGC--------CCTTCCCG...CTCCGTGACAGAAACCAACGGGCGT...CAAACTCACAACCCACTTGGC
C. ti	TCATCGATTC--------GCTTCCCG...CCTTGTGCCAGAAACCAACACACCT...CGAACCAAGAACCATCCTGGT
C. tropicale	TCATCGATTC--------CCTTCTCG...CCCTGCGCCAGAGACCAACGGGCCT...CGAACCAAGAATCATCCTGGT
C. viniferum	TCGTCGATTC--------ACTTCCCG...C-CTGCGCGAGAAACCAACAGACCT...GGAACCAAGAATCATCCTGGA
C. xanthorrhoeae	TCATCGATTC--------CCTTCCCG...CTCTGCGCCAGAAACCAACAGACCT...CAAATCAAGAACCATCCTGGT

	5' NOLADF 2 bp NOLADP 280 bp NOLADR 3'
C. noveboracense	GGGGAAGTA-TAGTCAGCGCATTG...CGTCATGACTGGAATTTGTGATGTTCC...GTCGAACGAACGAGAGACGGCGATTA
C. aenigma	AACGAGGGA-CTGTCAGCGAATTG...TGTCATGACTGGAGCTTCCGAGGTCTC...GTCGAACGAACGAGAGACGGCGATTA
C. camelliae	GACGAGAGAATTGTCAGCGCATTG...CGTCGTGACTGGAACTTCCGAGATCTG...GCCGAGCGAACGAGAGACGACAAATA
C. chrysophilum	GACGAGAGG-TTGTCAGCGCATTG...CATCGTGGCTGTAATTTTGGATGTTTC...ATCGAGCGAAGGAGAGACGACAAATC
C. fructicola	AACGAGGGA-CTGTCAGCGCATTG...TCTCATGACAGGAGCTTCCGAGATTTC...ACCGGGCGAAGGAGGGACGACAAATC
C. gloeosporioides	GACGAGAGG-TTGTCAGCGCATTG...CGTCATGACTGAAA-TTGTGGTGCTTC...ATCGAGCGAACGAGAGACGACGAGTC
C. nupharicola	GGGGAAGTA-TAGTCAGCGCATTG...CGTCATGACTGGAATTTGTGATGTTCC...GTCGAACGAACGAGAGACGGCGATTA
C. siamense	GACGAGAAG-TTGTCAGCGCATTG...CGTCATGACTGAAA-TTGTGGCGTTTC...ATCGACCGAACGAGAGACGACGAATC
C. theobromicola	GAGAGGGATGTTGTCAGCGCATTG...T-ACACATCAGGAGTTTCTCTTCTCGG...GTCGAACGAACGAGAGACGACGGATG
C. tropicale	GACGAGAAG-TTGTCAGCGCATTG...CGTCATGACTGAAA-TCGTGGCGTGTC...ATCGAGCGAAGGAGGGACGACGAATA
C. viniferum	AACAAGGGA-CTGTCAGCGGATTG...TGTCATGACTGGAGCTTCCGAGGTCTC...ATCGACCGAGCGAGAGACGATGAATC

	5' NYMGF 54 bp NYMGP 24 bp NYMGR 3'
C. nymphaeae	GATAACACCAGCTTCGTCGATAT--C...GATTGGGC---TTGTTGTAACGACACG...GCCGAGACAAAACTTGCTGACAGA
C. acutatum	GATAACACCAGCTTCGTCGGTAC--C...GATTGGGC---TCGTTGTAATGATGCG...GCCGAGACAAAATTTGCTGACAGA
C. cuscutae	GATAACACCAGCTTCGTCAATAT--C...GATTGGGC---TTGTTGTAACAACACG...GCCGAGACAAAACTTGCTGACAGA
C. fioriniae	AATAACACCAGCTTCATCGGTAA--C...GATTGGGCTCGTTGTTGTAATGATACG...GCCCAAACAAAATTTGCTGACAGA
C. kinghornii	ACGATAACACCACCCTACTCGGTAAC...GATTTGGC---TCGTTGCAATGGCACG...CCCAAGACAAAATTTGCTGACAGA
C. kniphofiae	GATAACACCACCCCAATCGGTTA--C...GATTTGGC---TTGTTGCAATGGCACC...TCCCAAGAAAAGATTTATTAACAG
C. lupini	GATAACACCAGCTTCGTCGGTAC--C...GATTGGGC---TTGTTGTAATGACACG...GCCGAGACAAAACTTGCTGACAGA
C. orientalis	AATAACACCAGCTTCATCGGTAA--C...GATTGGGCTCGTTGTTGTAATGATACG...GCCCAAACAAAATTTGCTGACAGA
C. pyricola	GATAACACCATCCTAATCGGTAA--C...GATTTGGC---TCGTTGCAATGGCAAG...TCCGAGACAAAATTTGCTGACAGA
C. scovillei	GATAACACCAGCTTCGTCGATAT--C...GATTGGGC---TTGTTGTAACGACACG...GCCGAGACAAAATTTGCTGACAGA

	5' SIAPF 1 bp SIAPP 96 bp SIAPR 3'
C. siamense	ACTGATATCGGCGCTGCCAG...CGACCTAAGGTTGTCTTTGTGTCCTAG...CACATCTGGCCATCGATTC--------CCTTC
C. aenigma	ATAGATATCGGCGCTGCCAG...CCACCTTAGGTCGTCCTTGTGTTCTAG...CGCATCTGGTCGTCGATTC--------ACTTC
C. aeschynomenes	ACCGATATCGGCGCTGCCAG...CGACCTCAGGTTGTCTTTGTGTCCTAG...CACATCTGGTCATCGATTC---------TTTG
C. alatae	ACCGATATCGGCGCTGTTAG...CGACATGAAGCTGTCTCTATGTCCTAG...CGCATCTGATCATCAGTTC--------CATTC
C. alienum	ATAGATATCGGCGCTGCCAA...CGACTTTAGGTCGTCCTTGTGTTCTAG...CGCATCTGGTCGTCGATTC--------ACTTC
C. aotearoa	ACCGATATCGGCGCTGCTAG...CGACATTAGGTTGTCTTTGTATCCTAG...AAGATCTGGTCATCGATTC--------GCTTC
C. camelliae	ACCGATATCGGCGCTGCTAG...CGACATTAGGTTGTCTTTGTATCCTAG...AAGATCTGGTCATCGATTC--------GCTTC
C. chrysophilum	ATAGATATCGGCGCTGCCAA...CGACTTTAGGTCGTCCTTGTGTTCTAG...CGCATCTGTTCGTCGATTC--------ACTTC
C. clidemiae	ACCGATATCGGCGCTGCTAG...CGACATTAGTTTGTCTTTGTATCCTAG...AAGATCTGGTCATCGATTC--------GCTTC
C. conoides	ATAGATATCGGCGCTGCCAG...CGACTTTAGGTCGTCCTTGTGTTCTAG...CGCATCTGTTCGTCGATTC--------ACTTC
C. fructicola	ATAGATATCGGCGCTGCCAA...CGACTTTAGGTCGTCCTTGTGTTCTAG...CGCATCTGTTCGTCGATTC--------ACTTC
C. gloeosporioides	ACCGATATCGGCGCTGCCAG...CCACCTTAGGTTGTCTTTGTGCCCTTG...TGCATCTGGTCATCGATTC--------CCTTC
C. henanense	ACCGATATCGACGCTACTTG...CGGCATCAGGTGGTCTTTTTATCCTAG...AACATCTGGTGACTTGGTCATCGATTCGCTTC
C. horii	ACCGATAGCGGCGCTGCTAG...CGACATGAGAATGTGTTTGTATCC-AG...CGCATCTGGTCGTCGATTC--------CCTTC
C. jiangxiense	ACCGATAGCGGCGCTGCTAG...CGACATTAGGTTGTCTTTGTATCCTAG...AAGATCTGGTCATCGATTC--------GCTTC
C. musae	ATAGATATCGGCGCTGCCAG...CGACTCTAGGTCGTCCTTGTGTTCTAG...CGCACCTGGTCGTCGATTC--------ACTTC
C. noveboracense	ATAGATATCGGCGCTGCCAA...CGACTTTAGGTCGCCCTTGTGTTCTAG...CGCATCTGTTCGTCGATTC--------ACTTC
C. perseae	ATAGATATCGGCGCTGCCAG...CGACTTTAGGTCGTCCTTGTGTTCTAG...CGCACCTGGTCGTCGATTC--------ACTTC
C. psidii	ACCGATATCGGCGCTGCTAC...CGACATTAGTTTGTCTTTGTATCCTAG...AAGATCTGGTCATCGATTC--------GCTTC
C. queenslandicum	ACCGATATCGACGCTGCCAG...CAACCTCAAGTTGTCTTTATGTCCTAG...CACATCTGGTCATCGATTC--------CCTTC
C. salsolae	ACTGATATCGGCGCTGCCAG...CGACCTCAGGTTGTCTTTGTGTCCTAG...CACATCTGGCCATCGATTT--------CCTTC
C. theobromicola	ACTAATGTCCGCGCAACTAG...CGACATTGGGTTGTCTTTGTATCCCAT...CGCCTCTGGCCAGCAACGC--------CCTTC
C. ti	ACCGATATCGGCGCTGCCAG...CGCCATTAGGTTGTCTTTGTATCCTAG...AAGATCTGGTCATCGATTC--------GCTTC
C. tropicale	ATCGACATCGGCGCTGCTAG...CGACCTCAGGCTGTCCTTGTGTCCTAG...CGCATCTGCTCATCGATTC--------CCTTC
C. viniferum	ATAGATATCGGCGCTGCCAG...CCACCTCAGGTCGTCCTTGTGTTCTAG...CGCATCTGGTCGTCGATTC--------ACTTC
C. xanthorrhoeae	ACCGATATCGGCGCTGCTAG...CGACATTGGGCTGTCTTTGTATCCTAG...CGGAGCTGGTCATCGATTC--------CCTTC

	5' THTUBF 14 bp THTUBP 106 bp THTUBR 3'
C. theobromicola	CTTTCACCCGAGTTCCATGTTCACC...CGTCAATC--CGACCCCCTACTGCG...GCAGGGCTAAGGGCTAATC--TCTCGC
C. aenigma	TTTTTACCCGACTTCTATGCACAAC...TGTCAATCATCGACGCCCAACTCTG...GCGGGG-------CTAACC--TCCTTG
C. aeschynomenes	TTTTTACCCGACTTCTATGCTCAAC...TGTCAATCATCGACGTCCAACTCTG...GCGGGG-------CTAACC--TCCTTG
C. alatae	TTTTTACCCGACTTCCATGCTCACC...CGTCAACCATCGACTTCCTACTCTG...GCGGGG-------CTAACC--TCCTTG
C. alienum	TTTTTACCCGACTTCTATGCACAAC...TGTCAATCATCGACGCCCAACTCTG...GCGGGG-------CTAACC--TCCTTG
C. aotearoa	GTTTTAC-------CCATGCTCACC...CGTCAATCATCGACCTCCTACTCTG...GCGGGG-------CTAACC--TCTTTG

C. asianum	**T**TTT**T**ACCCGA**C**TTCCATG**C**TCA**A**C...**T**GTCAATC**AT**CGACC**T**CC**A**ACT**CT**G...GC**G**GGG-------CTAA**C**C--TC**CTTG**
C. camelliae	**G**TTT**T**AC-------CCATG**A**TCACC...CGTCAATC**AT**CGACC**T**CCTACT**CT**G...GC**G**GGG-------CTAA**C**C--TCT**TTG**
C. changpingense	**T**TTT**T**ACCCGA**C**TTC**T**ATG**C**TCA**A**C...**T**GTCAATC**AT**CGAC**GT**CC**A**ACT**CT**G...GC**G**GGG-------CTAA**C**C--TC**CTTG**
C. chrysophilum	**T**TTT**T**ACCCGA**C**TTC**T**ATG**CA**CA**A**C...**T**GTCAATC**AT**CGAC**T**CCC**A**ACT**CT**G...GC**G**GGG-------CTAA**C**C--TC**CTTG**
C. cigarro	**G**TTT**T**AC-------CCATG**C**TCACC...CGTCAATC**AT**CGACC**T**CCTACT**CT**G...GC**G**GGG-------CTAA**C**C--TCT**TTG**
C. cordylinicola	**G**TTT**T**AC-------CCATG**C**TCACC...CGTCAATC**AT**CGACC**T**CCTACT**CT**G...GC**G**GGG-------CTAA**C**C--TCT**TTG**
C. endophyticum	**T**TTT**T**ACCCGA**C**TTCCATG**C**TCA**A**C...**T**CTCAATC**AT**CGACC**T**CCTACT**C**CG...GC**G**GGG-------CTAA**C**C--TC**CTTG**
C. fructicola	**T**TTT**T**ACCCGA**C**TTC**T**ATG**CA**CA**A**C...**T**GTCAATC**AT**CGAC**G**CCC**A**ACT**CT**G...GC**G**GGG-------CTAA**C**C--TC**CTTG**
C. gloeosporioides	**T**TTT**T**ACCCGA**CC**TC**TT**TG**C**TCA**A**C...**T**GTCAATC**AT**CGACC**T**CCTA**G**T**CT**G...GC**G**GGG-------CTAA**C**C--TC**CTTG**
C. grevilleae	CTTTCACCCGAGTTCCATGTTCACC...CGTCAATC--CGACCCCCTACTGCG...GCAGGG-------CTAATC--TCTCGC
C. grossum	CTTTCACCCGAGTTCCATGTTCACC...CGTCAATC--CGACCCCCTACTGCG...GCAGGG-------CTAATC--TCTCGC
C. henanense	**G**TTT**T**AC-------CCATG**C**TCACC...CGTCAATC**AT**CGACC**T**CCTACT**CT**G...GC**G**GGG-------CTAA**C**C--TCT**TTG**
C. horii	**T**TTT**T**ACCCGA**C**TTCCATG**C**TCA**A**C...C**T**TCAATT**AT**CGAC**GT**CCTACT**CT**G...GC**G**GGG-------CTAA**C**C**AGAG**T**TCT**
C. jiangxiense	**G**TTT**T**AC-------CCATG**C**TCACC...CGTCAATC**AT**CGACC**T**CCTACT**CT**G...GC**G**GGG-------CTAA**C**C--TCT**TTG**
C. kahawae	**G**TTT**T**AC-------CCATG**C**TCACC...CGTCAATC**AT**CGACC**T**CCTACT**CT**G...GC**G**GGG-------CTAA**C**C--TCT**TTG**
C. musae	**T**T-------------ATG**CA**CA**A**C...**T**GTCAATC**AT**CGAC**G**CCC**A**ACT**CT**G...GC**G**GGG-------CTAA**C**C--TC**CTTG**
C. noveboracense	**T**TTT**T**ACCCGA**C**TTC**T**ATG**CA**CA**A**C...**T**GTCAATC**AT**CGAC**G**CCC**A**ACT**CT**G...GC**G**GGG-------CTAA**C**C--TC**CTTG**
C. proteae	**T**TTT**G**ACCCGA**C**TTCCATG**C**TCA**A**C...**T**GTCAATC**AT**CGAC**T**CC**A**TACT**CT**G...GC**G**GGG-------CTAA**C**C--TC**CGTG**
C. queenslandicum	**T**TTT**T**ACCCGA**C**TTCCATG**C**TCA**A**C...**T**GTCAATC**AT**CGAC**GT**CC**A**ACT**CT**G...GC**G**GGG-------CTAA**C**C--TC**CTTG**
C. salsolae	**T**TTT**T**ACCCGA**C**TTC**T**ATG**C**TCA**A**C...**T**GT**T**AATC**AT**CGAC**GT**CC**A**ACT**CT**G...GC**G**GGG-------CTAA**C**C--TC**CTCG**
C. siamense	**T**TTT**T**ACCCGA**C**TTC**T**ATG**C**TCA**A**C...**T**GTCAATC**AT**CGAC**GT**CC**A**ACT**CT**G...GC**G**GGG-------CTAA**C**C--TC**CTTG**
C. tropicale	**T**TTT**T**ACCCGA**T**TTC**T**ATG**C**TCA**A**C...**T**GTCAATC**AT**CGAC**GT**CC**A**ACT**CT**G...GC**G**GGG-------CTAA**C**C--TC**CTTG**
C. viniferum	**T**TTT**T**ACCCGA**C**TTC**T**ATG**CA**CA**A**C...**T**GTCAATC**AT**CGAC**G**CC**AA**ACT**CT**G...GC**G**GGG-------CTAA**C**C--TC**CTTG**
C. wuxiense	**G**TTT**T**AC-------CCATG**A**TCACC...CGTCAATC**AT**CGACC**T**CCTACT**CT**G...GC**G**GGG-------CTAA**C**C--TCT**TTG**

Figure S2. Alignments of available *Colletotrichum* accessions at primer and probe sites for each primer-probe set. **Bold** indicates mismatches with the target species, "-" is a gap, and "…" is excluded sequence. For *C. chrysophilum* ladA, *C. fructicola* ladA, C. *gloeosporioides* GAPDH, *C. noveboracense* ladA, *C. nymphaeae* GAPDH, and *C. theobromicola* THTUB, accessions outside their species complex were excluded due to high sequence dissimilarity at these primer and probe sites.
